# Comparison of Volatiles Profile and Contents of Trichothecenes Group B, Ergosterol, and ATP of Bread Wheat, Durum Wheat, and Triticale Grain Naturally Contaminated by Mycobiota

**DOI:** 10.3389/fpls.2016.01243

**Published:** 2016-08-22

**Authors:** Maciej Buśko, Kinga Stuper, Henryk Jeleń, Tomasz Góral, Jarosław Chmielewski, Bożena Tyrakowska, Juliusz Perkowski

**Affiliations:** ^1^Department of Chemistry, Poznań University of Life SciencesPoznan, Poland; ^2^Department of Food Science and Nutrition, Poznań University of Life SciencesPoznan, Poland; ^3^Department of Plant Pathology, Plant Breeding and Acclimatization Institute NRIRadzików, Poland; ^4^Department of Instrumental Analysis, Poznań University of EconomicsPoznan, Poland

**Keywords:** volatile organic compounds, trichothecenes, ergosterol, ATP, bread wheat, durum wheat, triticale

## Abstract

In natural conditions cereals can be infested by pathogenic fungi. These can reduce the grain yield and quality by contamination with mycotoxins which are harmful for plants, animals, and humans. To date, performed studies of the compounds profile have allowed for the distinction of individual species of fungi. The aim of this study was to determine the profile of volatile compounds and trichothecenes of group B, ergosterol, adenosine triphosphate content carried out on a representative sample of 16 genotypes of related cereals: triticale, bread wheat, and durum wheat. Based on an analysis of volatile compounds by means of gas chromatography mass spectrometry and with the use of an electronic nose, volatile profiles for cereals were determined. Differentiation is presented at four levels through discriminant analysis, heatmaps, principal component analysis (PCA), and electronic nose maps. The statistical model was built by subsequent incorporation of chemical groups such as trichothecenes (GC/MS), fungal biomass indicators ergosterol (HPLC) and ATP (luminometric) and volatiles. The results of the discriminatory analyses showed that the volatile metabolites most markedly differentiated grain samples, among which were mainly: lilial, trichodiene, p-xylene. Electronic nose analysis made it possible to completely separate all the analyzed cereals based only on 100 ions from the 50–150 m/z range. The research carried out using chemometric analysis indicated significant differences in the volatile metabolites present in the grain of bread wheat, durum wheat and triticale. The end result of the performed analyses was a complete discrimination of the examined cereals based on the metabolites present in their grain.

## Introduction

Cereals are a staple food in human nutrition and are also the most important materials in farming for animal feeds (Condain, 1999). Several species of cereals are grown, among which wheat cultivation plays a crucial role. Despite genetic similarities, well known cultivated varieties of wheat differ from each other, viz.: durum wheat [*Triticum turgidum* L. subsp. *durum* (Desf.) Husn.] is a tetraploid species with genome including an A genome of *Triticum urartu* and a B genome of probably *Aegilops speltoide*s. Bread wheat (*Triticum aestivum* L. subsp. *aestivum*) is a hexaploid species with genome including an AB genome of durum wheat and a D genome of *Aegilops tauschii* Coss (Chantret et al., 2005). Cultivated hexaploid triticale (X *Triticosecale* Wittmack), is an artificially created cereal, resulting from the crossing of wheat (*T. aestivum* L. subsp. *aestivum*) or durum wheat (*T. turgidum* L. subsp. *durum*) with rye (*Secale cereale* L.); this includes the A and B genomes of *Triticum* and R of *Secale* (Eudes, 2015). Cereals also differ in their susceptibility to fungal diseases.

In view of food safety, it is important to monitor the microbial status of grain. In natural conditions cereals can be infested by numerous pathogens, of which fungi are among the most important. Some of them, such as fungi from the genus *Fusarium*, can cause devastating diseases. These lead to severe economic losses by reducing the grain yield and quality by contamination with mycotoxins which are harmful not only for plants but also animals and humans.

Among the mycotoxins groups described in the literature much attention is focused on a small number of mycotoxins due to their high frequency of occurrence. These include trichothecenes group B with the most commonly occurring deoxynivalenol (DON) (Stępień and Chełkowski, 2010). Trichothecene biosynthesis by fungi is controlled by many factors. The main factor is genetic, determining both, the quantity and quality of created toxins. Related to this is the effect of the different chemotypes of fungi (Lee et al., 2002; Kulik, 2011; Mugrabi de Kuppler et al., 2011) which are often regional in nature (Logrieco and Visconti, 2004). Factors influencing the formation of trichothecenes are: substrate, the time of infection, competition between microorganism, plant-pathogen interaction, and overall climatic conditions among which temperature, humidity, and rainfall are the most important (Champeil et al., 2004). The complexity of the trichothecenes biosynthesis is one of the reasons for the controversy regarding their participation in pathogenesis because trichothecenes are not essential for infection (Hestbjerg et al., 2002). However, some studies have shown that trichothecenes contribute to the virulence of *Fusarium* (Proctor et al., 2002; Ayumi and Manickavelu, 2015), mainly due to the inhibition of protein synthesis (Mitterbauer et al., 2004). All this information, together with the fact that in the case of various types of cereals we have to deal with the diversity of their resistance to FHB, means that descriptions of new types of resistance are often observed. The influence of trichothecenes on FHB is related to the second type of resistance described for wheat (Schroeder and Christensen, 1963) and resistance to the accumulation of trichothecenes through metabolic transformation (Boutigny et al., 2008).

Metabolomics, a relatively new field of knowledge, provides more and more opportunities to examine the plant metabolome, including cereals (Balmer et al., 2013). An extremely important aspect of these studies is connected with analyses of metabolomic profiles of microorganisms, particularly pathogenic, and toxigenic (Panagiotou et al., 2005), with a significant role being played by those metabolites which, being formed in grain, are frequently precursors for the formation of others (Perkowski et al., 2008a). Of these the most frequently analyzed metabolites to date are ergosterol (ERG) (Müller and Schwadorf, 1999; Perkowski et al., 2008b) and to a lesser extent ATP (Suberkropp et al., 1993; Perkowski et al., 2007).

ERG analysis as the primary sterol of fungal cell membranes has proven useful with respect to both the native fungal biomass and dead. The latter is particularly important in the case of infection by toxigenic fungi such as *Fusarium* whose products, known as fusariotoxins, are typically very stable and remain in plant tissues, including the grain even in the absence of the longer-lived hyphae of the fungus. In a previous work also by the authors of this paper, it was shown that especially in conditions of massive infection, which takes place during the inoculation of plant with fungus, there is a significant correlation between the concentration of the ERG, and the concentration of trichothecenes (Perkowski et al., 2007). However, in conditions of natural infestation, which highlight the role of other factors, particularly agro-meteorological and the defense mechanisms of plants, the obtained values of correlation coefficients of ERG with trichothecenes often proved to be statistically insignificant. The use of ATP measurements to assess the amount of fungal biomass in the seed is not widespread, although it is a commonly applied method in industry and environmental studies. Essentially it is possible to isolate the ATP from the surface of grains and determine the level of total living biomass. Work conducted previously, particularly in inoculation experiments has revealed a significant correlation of surface ATP levels, with both the concentration of the ERG and trichothecenes (Perkowski et al., 2007).

From the numerous (metabolomic) studies on this subject it seems advisable to select the metabolic pathway proposed by Magan and Evans (2000) as the basis for discussion. This indicates the relationships between individual groups of compounds, i.e., volatiles, non-volatiles, fungal biomass indicators and mycotoxins, and shows volatile compounds to have an inhibitory effect on the growth of other microscopic fungi (Mellon et al., 2002, 2005).

The study of the volatile compound profile allowed for the distinction of individual species of fungi from the genera *Penicillium* and *Aspergillus* (Sahgal et al., 2007). Moreover, analyses were also conducted on profiles of volatile compounds formed by fungi from the genus *Fusarium* (Borjesson et al., 1992; Jeleń and Wąsowicz, 1998; Schnürer et al., 1999; Demyttenaere et al., 2003) From the entire spectrum of volatile compounds produced by these fungi, we particularly need to focus on trichodiene, since, being a secondary metabolite, it is considered as a precursor in the trichothecene biosynthesis pathway (Kimura et al., 2003; Perkowski et al., 2008a). Examinations of metabolites which are considered as fungal biomass markers—ERG and ATP are also of great importance. What is most interesting are the results that indicate a correlation between these markers and mycotoxins (Borjesson et al., 1992; Perkowski et al., 2007) and their relationship with volatile compounds (Demyttenaere et al., 2004; Sahgal et al., 2007; Girotti et al., 2012; Buśko et al., 2014).

The observations presented above and studies conducted to date confirm the applicability of analyses of volatile compounds in the classification of grain depending on the degree of infestation by fungi or even the determination of grain contaminated with mycotoxins. Efforts to accomplish the above-mentioned goals were also conducted by means of a set of sensors called an electronic nose (Borjesson et al., 1996). However, under natural conditions the volatiles profile of grain consists of metabolites of kernel as well as microbiota living on its surface. Thus, the examination of volatile organic compounds requires the appropriate natural conditions.

The aim of this study was to determine the VOCs and concentration of trichothecenes group B and mycobiota (ERG, ATP) in a representative sample of 16 genotypes of related cereals: triticale, bread wheat, and durum wheat. Special emphasis was placed on the performance of their comprehensive analysis as well as on an attempt to define the relationship between these parameters. Another objective was to determine the individual compounds differentiating grain of each cereal using chemometric analysis. Such a study in this respect, covering chemical analyses using different techniques (GC/MS, HPLC, luminometric, and the electronic nose), as well as analysing a large population of samples grown at the same time and under identical conditions, both climatic and agronomic, has not been presented to date.

## Materials and methods

In order to distinguish the analyzed cereals thoroughly, a moving statistical analysis was conducted based on metabolites present in cereals. The model allows for the subsequent implementation of different variables representing particular groups of chemical compounds such as *Fusarium* toxins, ergosterol (ERG), ATP and volatile compounds (VOCs).

### Material

Analyses were conducted on representative naturally contaminated samples of grain: spring triticale (16 genotypes), bread wheat (16 genotypes), and durum wheat (16 genotypes).

The genotypes comprised the most important cultivars regionalized in Poland—tested in state-commissioned trials.

Samples of bread wheat, triticale, and durum wheat were sown in a field experiment at the Plant Breeding and Acclimatization Institute in Radzików (central Poland) (GPS coordinates: 52.211754, 20.631954). Bread wheat and triticale were cereal varieties listed in the Polish National List of Agricultural Plant Varieties drawn up by the Research Centre for Cultivar Testing (COBORU http://www.coboru.pl). The sample of durum wheat was the only variant located in the registry (in the National List) and 15 advanced breeding lines.

Field experiments were established in a randomized block design. The cereals were sown in plots with an area of 10 m^2^, in triplicate. No fungicides were used during cultivation. Plants were grown and harvested in accordance with good agricultural practice standards under identical environmental conditions.

After ripening (BBCH 89), 500 heads were harvested manually from each plot and threshed with a laboratory thresher at a low wind speed to prevent loss of low-weight infected kernels. The percentage of damaged kernels was assessed visually (results not presented in this work). Grain samples of three reps (an aliquot of grains of 1 kg) were obtained. Immediately after harvest the profile of volatile compounds was analyzed together with the concentration of ERG, ATP, and trichothecenes. The results are expressed as the dry weight of the grain.

### Analysis of VOCs

Analysis of VOCs was performed as described by Buśko et al. (2010). VOCs were extracted from grain using the solid-phase microextraction method (SPME). Grain samples (8 g) were placed in 20 ml vials and extracted by means of headspace SPME for 30 min at 50°C with 200 mm −53/30 μm divinylbenzene/Carboxene/polydimethylsiloxane StableFlexTM (DVB/Carboxen/PDMS) fiber. The analyses were run on a gas chromatograph (Agilent 7890A) hyphenated to a mass spectrometer (TruTOF HT, LECO), using an RTX-5 (0.20 mm × 10 m) capillary column. The injection port temperature was 260°C, the transfer line temperature was 280°C and the analyses were performed with a programmed temperature: initial 40°C held for 1 min, from 40°C to 180°C at 10°C/min, 180 to 260°C at 40°C/min. The helium flow rate was held constant at 0.8 ml/min. Spectra were acquired at 50 spectra/s within a range of 30–380 Da. The detector voltage was 2500 V, electron energy 70 eV. The amount of VOCs was estimated by comparing the area of their total ion current (TIC) peaks with the internal standard (addition of 25 ng of tridecane in pentane) and expressed as their ratio (RU). Compounds were identified by comparing their mass spectra with spectra from the NBS 75K and NIST 98 libraries and retention indices were compared to data available in the literature. Retention indices were calculated based on a chromatographic analysis of chain alkanes (C4-C20)

### Electronic nose

The electronic nose was used to analyse mixtures of volatile substances, characterizing samples, and facilitating their identification as described in Perkowski et al. (2012). They were identified by comparing sample characteristics with the matrix recorded in the computer memory. The procedure for the application of the “electronic nose” is based on the direct analysis of a mixture of volatile compounds collected from samples with the headspace technique, transferred from the automatic injection port in a quadrupole mass spectrometer. In the headspace technique, by applying optimal conditions (temperature and time), the gas phase is obtained, whose composition in terms of quantity and quality reflects the composition of the original sample. Analyses were performed with a Perkin-Elmer apparatus, an HKR Sensorsysteme with TM SOFT NT and QMB SOFT NT software by HKR Sensorsysteme, using statistical discriminatory analysis to facilitate differentiation of products and graphic presentation of results. In all cases the analysis of a sample was performed in at least 10 replications, making it possible to obtain a mean value, most accurately corresponding to the analyzed sample.

### Analysis of trichothecenes

Samples of grain were analyzed according to Perkowski et al. (2003). Briefly, samples (10 g) were extracted with acetonitrile/water (82:18) and cleaned-up on a charcoal column [Celite 545/charcoal Draco G/60/activated alumina neutral 3:9:5 (w/w/w)].

Trichothecene toxins of group B (DON, 3-AcDON, 15-AcDON, FUS X, NIV; Sigma, St. Louis, USA) after derivatization with mixture of trimethylsilylimidazole—trichlorosilane (100–1 v/v) were analyzed by a mean gas chromatograph (Hewlett Packard GC 6890) coupled to a mass spectrometer (Hewlett Packard 5972 A, Waldbronn, Germany), using an HP-5MS, 0.25 mm × 30 m capillary column. The samples were injected into the injection port (at 250°C), the transfer line temperature was 280°C and the analyses were performed with the programmed temperature: initial temperature 80°C, held 1 min, from 80°C to 200°C at 15°C/min held 6 min and from 200°C to 280°C at 10°C/min, the final temperature being maintained for 3 min. The helium flow rate was constant at 0.7 ml/min. Quantitative analysis was performed with the external standard method in single ion monitored mode (SIM) using the following ions for the detection of DON: 103 and 512; 3-AcDON: 117 and 482; 15-AcDON: 193 and 482; FUS X: 103 and 570; NIV: 191 and 600. Recovery for analyzed toxins was as follows: DON, 84 ± 3.8%; 3AcDON, 78 ± 4.8%; 15Ac-DON, 74 ± 2.2%; FUS X, 87 ± 5.9%; NIV, 81 ± 3.8%. The limit of detection was 0.01 μg/kg. Qualitative analysis was performed in SCAN mode (100–700 amu).

### Analysis of ergosterol

ERG was determined by HPLC as described by Perkowski et al. (2008b). Samples containing 100 mg of ground grains were placed into 17-ml culture tubes, suspended in 2 ml of methanol, treated with 0.5 ml of 2 M aqueous sodium hydroxide, and tightly sealed. The culture tubes were then placed within 250-ml plastic bottles, tightly sealed and placed inside a microwave oven (Model AVM 401/1WH, Whirlpool, Sweden) operating at 2450 MHz and 900 W maximum output. Samples were irradiated (370 W) for 20 s and after ~5 min for an additional 20 s. After 15 min the contents of the culture tubes were neutralized with 1 M aqueous hydrochloric acid, 2 ml MeOH were added and extraction with pentane (3 × 4 ml) was carried out within the culture tubes. The combined pentane extracts were evaporated to dryness in a nitrogen stream. Before analysis samples were dissolved in 4 ml of MeOH, filtered through 13-mm syringe filters with a 0.5 μm pore diameter (Fluoropore Membrane Filters, Millipore, Ireland) and evaporated to dryness into an N_2_ stream. The sample extract was dissolved in 1 ml of MeOH and 50 μl were analyzed using an HPLC technique. Chromatographic separation was done on a 150 × 3.9 × 4 mm Nova Pak _C−18_ column and eluted with methanol/acetonitrile (90:10) at a flow rate of 0.6 ml/min. Detection was performed by means of a Waters 486 Tunable Absorbance Detector (Milford, MA, USA) set at 282 nm. Conformation of the presence of ERG was based on a comparison of retention times and the co-injection of every tenth sample with an ERG standard. The limit of detection was 0.02 mg/kg for the method.

### Analysis of ATP

Determination of ATP was performed according to Perkowski et al. (2012). ATP was extracted from the surface of a 1.00 g sample of kernels, using boiling tris buffer (8 ml, 0.1 M solution, pH-7.75). After which 100 μl of the cooled extract was added to 100 μl reagent [luciferin and luciferase eLuminATE (QM) from the Microbial Biomass kit supplied by Celsis, Netherlands]. The light emission was measured by a Lumac Biocounter M 1500 luminometer and read as Relative Light Units (RLU). The emitted light correlates with the amount of ATP in the sample as reported by Suberkropp et al. (1993).

### Statistical analysis

The results recorded in the course of the conducted chemical analyses were subjected to statistical analysis with the use of STATISTICA ver. 8.0 (StatSoft, Inc., Tulsa, OK, USA) and Microsoft® Excel 2010/XLSTAT©-Pro Version 2015.1.02 (Addinsoft, Inc., Brooklyn, NY, USA) software packages. In order to compare contents of volatile compounds in samples Tukey's multiple comparison procedure was used, with identical letters in rows or columns denoting a lack of differences at the significance level *P* = 0.05. Moreover, a step linear discriminatory analysis (SLDA) and the principal component analysis (PCA) were used in order to separate groups of analyzed populations. Additionally, the classification matrix and multivariate significance tests were applied. On the basis of cluster analysis heatmaps were prepared, with marked regions differentiating analyzed groups at the confidence level *P* = 0.05.

## Results

**Figure 1:**
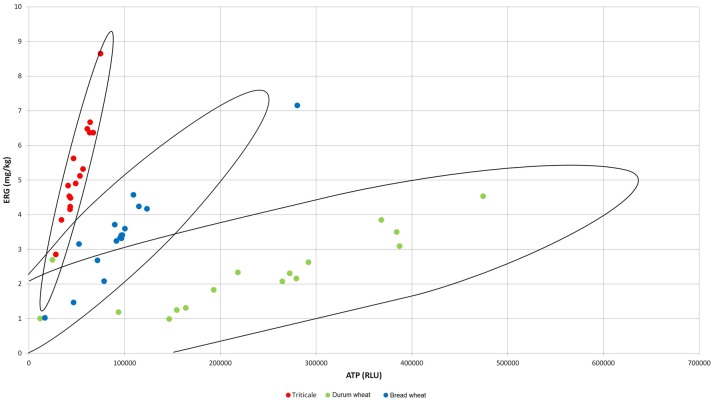
**Correlation between ERG concentration (mg/kg) and ATP level (RLU) for triticale, bread wheat, and durum wheat**.

The significantly highest concentration of ERG was determined in triticale, it was lower in bread wheat and the lowest in durum wheat (Table 1). The second indicator of microbial contamination of grain was ATP, which only allows the level of the surface contamination with living cells to be determined. The ATP levels were found to be significantly different only for durum wheat. Bread wheat and triticale did not significantly differ from each other in terms of ATP concentrations as shown in Table 1. The determined concentration of group B trichothecene mycotoxins is shown in Table 1. The concentration of the sum was highest for durum wheat, with <50% for bread wheat and approx. 70% for triticale relative to durum wheat, the only significant differences were observed between durum wheat and triticale. Among the four analyzed mycotoxins DON was found to have the largest share in the total concentrations of trichothecenes for durum wheat, 75% and bread wheat up to 82%, the highest concentration being respectively 116.94 μg/kg and 55.69 μg/kg. The concentration of acyl derivatives of DON in trials in/on cereals was low and not statistically different. As indicated above, the lowest level of tested DON was found for triticale, although it was characterized by a five-fold higher concentration of 3-AcDON than the other cereals. Accordingly, it is the 3-AcDON toxin that was dominant and the concentration of DON here was more than 30% lower than the concentration of 3-AcDON. The highest concentration of NIV was determined in the durum wheat, the toxin being identified in 81% of the trials. The analyzed NIV concentration in the other two species of cereals was nearly eight-fold lower, and designated in 56% of trials. Based on the results of chemical analyses, a correlation analysis was performed (Table 2). Obtained correlation matrices showed the greatest contribution to the correlation of toxins in the case of the entire population of samples, and an inversely proportional correlation was revealed between the concentration of toxins, and the concentration of the ERG. The highest correlation with concentrations of toxins summary for all tested species of cereals was the predominant toxin—DON. However, highly significant correlations were also observed in the case of cereals particularly when considered separately (Figure 1). An example is that the means of all the analyzed cereals differ significantly in ERG/ATP ratio. This is due to the fact that the level of contaminating mycobiota was characteristic for individual cereal species. This indicates that a highly significant correlation coefficient exists for ERG and ATP: for a single species of triticale grain, respectively: 0.947^***^; durum wheat: 0.810^***^; wheat bread: 0.932^***^, but if the entire population of grain was not negligible.

**Table 1:** **Mean concentration of trichothecenes of group B (μg/kg), ergosterol (mg/kg), and level of ATP (RLU) in 16 genotypes of triticale, durum wheat, and bread wheat**.

**Compound**	**Triticale**			**Durum wheat**			**Bread wheat**		
	**Mean**	**SE**	**Positive samples**	**Mean**	**SE**	**Positive samples**	**Mean**	**SE**	**Positive samples**
DON	16.98^a^	6.34	94	116.94^b^	38.12	94	55.69^ab^	24.12	81
3-AcDON	24.76^b^	3.05	100	5.29^a^	1.58	50	4.68^a^	0.98	81
15-AcDON	0.38^a^	0.18	25	0.40^a^	0.15	31	2.02^a^	0.88	56
NIV	4.61^a^	1.31	56	32.32^b^	10.92	81	5.12^a^	1.60	56
SumTox	46.74^a^	8.51		154.95^b^	44.21		67.51^ab^	24.53	
ERG	5.28^c^	0.35		2.30^a^	0.26		3.41^b^	0.35	
ATP	50,744^a^	3211		23,3015^b^	33,123		97,605^a^	13,961	

*Values in separate columns marked with the same letter (a, b, c) do not differ significantly at p = 0.05, according to HSD Tukey test*.

*SE—standard error, positive samples—%*.

**Table 2:** **Coefficients of Pearson correlations between ATP, ERG, and trichothecene mycotoxins for three cereals (***n*** = 16) and for all (***n*** = 48) analyzed cereals samples (Total)**.

	**ATP**	**ERG**	**DON**	**3-AcDON**	**15-AcDON**	**NIV**
**TRITICALE**						
ERG	0.947^***^					
DON	0.438	0.253				
3-AcDON	0.430	0.367	0.347			
15-AcDON	−0.017	0.105	0.103	0.708^**^		
NIV	0.474	0.404	0.253	0.374	0.284	
SumTox	0.553^*^	0.380	0.906^***^	0.687^**^	0.242	0.482
**DURUM WHEAT**						
ERG	0.810^***^					
DON	0.084	−0.110				
3-AcDON	0.060	−0.182	−0.122			
15-AcDON	0.142	0.044	0.060	0.209		
NIV	0.475	−0.138	0.488	−0.343	−0.179	
SumTox	0.187	−0.135	0.979^***^	−0.154	0.019	0.655^**^
**BREAD WHEAT**						
ERG	0.932^***^					
DON	−0.336	−0.393				
3-AcDON	−0.369	−0.458	0.497			
15-AcDON	−0.214	−0.391	−0.035	0.164		
NIV	−0.189	−0.240	−0.098	0.027	0.164	
SumTox	−0.365	−0.435	0.996^***^	0.543^*^	0.027	−0.024
**TOTAL SAMPLES**						
ERG	−0.121					
DON	0.171	−0.353^*^				
3-AcDON	−0.349^*^	0.528^***^	−0.180			
15-AcDON	−0.102	−0.201	−0.026	−0.001		
NIV	0.033	−0.293^*^	0.488^***^	−0.236	−0.078	
SumTox	0.123	−0.331^*^	0.979^***^	−0.114	−0.023	0.634^***^

* Value significant at p = 0.05;

** value significant at p = 0.01;

*** *value significant at p = 0.001*.

**Figure 2:**
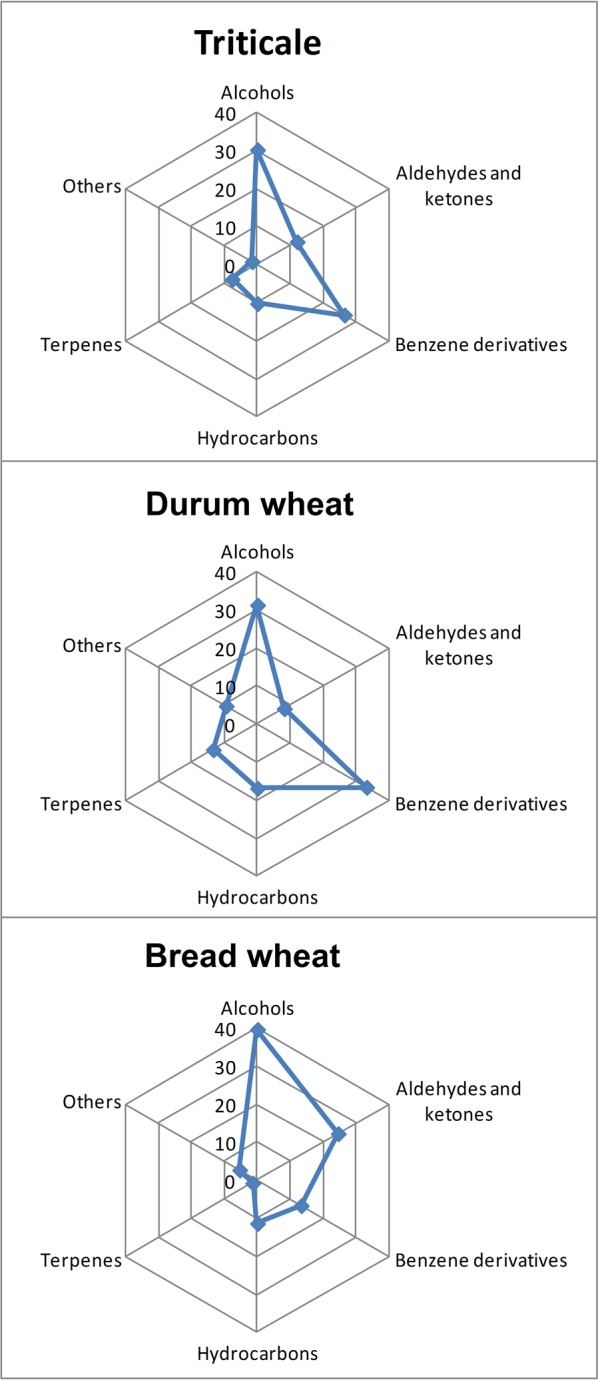
**Mean percentage contents of volatile compounds differentiating triticale, bread wheat, and durum wheat**.

The above-mentioned metabolites account for a further discrimination of the examined cereals. Microscopic fungi produce a variety of volatile compounds through their metabolism. Analysis of the presence of volatile compounds in the examined cereals revealed a broad spectrum of VOCs, comprising over 1600 signals. These signals (a combination of the retention index and unique ions) were found in different cereals at different intensities. Of these, 201 were chosen, whose incidence in individual grain samples exceeded 40%, as shown in Figure 2. A total of 46 volatile compounds were identified (Table 3). These 46 compounds were detected in all analyzed samples of particular cereals. They belonged to five different chemical groups—alcohols, aldehydes and ketones, benzene derivatives, hydrocarbons, and terpenes—and also to a sixth group, designated as “others”—butyrolactone, dimethyl sulfone, octanoic acid, 5-butyldihydro-2-(3H)-furanone, nonanoic acid, and diethyl phthalate (Table 3). The most numerous compounds were alcohols. This group of volatiles was present in the highest amount in bread wheat, as presented in Figure 2, although a significant difference was found only between bread wheat and triticale (Table 3). The next most abundant group comprised benzene derivatives, amounts of which were significantly different between particular cereals; the highest amount was found in durum wheat, followed by triticale, and the lowest in bread wheat (Figure 2). Significantly (two-fold) higher levels of aldehydes and ketones were observed in samples of bread wheat. In the case of hydrocarbons the highest percent content was noted for durum wheat, but this did not differ significantly from bread wheat. Although the lowest share among volatile compounds was found for terpenes, their percentages for all three cereals were statistically different (Table 3).

**Table 3:** **Qualitative and quantitative (RU) profile of 46 volatile metabolites for 16 genotypes of triticale, durum wheat, and bread wheat**.

**Lp**	**RI**	**Compound**	**Triticale**	**Durum wheat**	**Bread wheat**	**Metabolite group**	**Wilk's lambda**	**F displacement**
1	870	p-Xylene^*^	1.486^c^	0.539^b^	0.283^a^	Benzene derivatives	0.237	5.964
2	873	1-Hexanol	3.512^a^	5.210^b^	3.732^a^	Alcohols		
3	889	Styrene	0.173^a^	0.077^a^	0.046^a^	Benzene derivatives		
4	901	Heptanal^*^	0.328^a^	0.392^a^	0.561^b^	Aldehydes and ketones	0.460	1.984
5	916	Butyrolactone	0.690^a^	1.529^b^	0.815^a^	Others		
6	927	Dimethyl sulfone	0.001^a^	0.000^a^	0.000^a^	Others		
7	929	Alpha-Pinene	0.158^a^	0.611^b^	0.067^a^	Terpenes		
8	948	Propylbenzene	0.450^a^	0.926^b^	0.437^a^	Benzene derivatives		
9	957	1-ethyl-2-methylbenzene	1.289^a^	3.422^b^	0.956^a^	Benzene derivatives		
10	963	1.3.5-trimethylbenzene	0.299^a^	0.668^b^	0.288^a^	Benzene derivatives		
11	969	1-heptanol^*^	0.173^a^	0.181^a^	0.221^a^	Alcohols	0.245	3.156
12	974	1-ethyl-3-methyl-benzene^*^	0.170^a^	0.458^b^	0.164^a^	Benzene derivatives	0.879	1.174
13	978	1-octen-3-ol^*^	0.199^a^	0.303^b^	0.248^a^	Alcohols	0.261	3.127
14	985	6-methyl-5-Hepten-2-one	0.164^a^	0.166^a^	0.220^a^	Aldehydes and ketones		
15	988	Benzene	1.211^a^	2.696^b^	1.060^a^	Benzene derivatives		
16	989	2-octanone	0.161^a^	0.335^b^	0.321^b^	Aldehydes and ketones		
17	1005	3-carene^*^	0.087^a^	0.123^b^	0.062^a^	Terpenes	0.554	1.845
18	1020	Cymene	0.064^a^	0.185^b^	0.053^a^	Terpenes		
19	1024	Limonene	0.187^a^	0.750^b^	0.165^a^	Terpenes		
20	1029	2-ethyl-1-hexanol	3.695^a^	3.403^a^	3.365^a^	Alcohols		
21	1030	Indane^*^	0.037^a^	0.056^a^	0.026^a^	Benzene derivatives	0.407	2.369
22	1038	3-octen-2-one	0.065^a^	0.082^a^	0.071^a^	Aldehydes and ketones		
23	1092	Nonan-2-one	0.027^a^	0.039^a^	0.147^b^	Aldehydes and ketones		
24	1094	(E.E)-3.5-Octadien-2-one	0.005^a^	0.031^a^	0.042^a^	Aldehydes and ketones		
25	1099	Undecane^*^	0.183^a^	0.442^c^	0.205^b^	Hydrocarbons	0.404	2.554
26	1105	Nonanal	0.761^b^	0.748^b^	1.523^a^	Aldehydes and ketones		
27	1178	Naphthalene	0.450^b^	0.361^a^	0.392^a^	Benzene derivatives		
28	1192	3.5.5-Trimethylcyclohexen-2-one	0.007^a^	0.000^a^	0.059^a^	Aldehydes and ketones		
29	1192	Octanoic Acid	0.183^a^	0.518^b^	0.148^a^	Others		
30	1199	Dodecane	0.278^a^	0.425^b^	0.251^a^	Hydrocarbons		
31	1206	Decanal	0.258^a^	0.146^a^	0.822^b^	Aldehydes and ketones		
32	1221	2-phenoxyethanol^*^	0.211^a^	0.352^a^	0.382^a^	Alcohols	0.315	2.493
33	1261	5-butyldihydro-2(3H)-furanone	0.084^a^	0.097^a^	0.112^a^	Others		
34	1276	1-decanol^*^	0.099^a^	0.337^c^	0.261^b^	Alcohols	0.349	5.871
35	1283	Nonanoic acid	0.112^a^	0.164^a^	0.160^a^	Others		
36	1291	2-methylnaphthalene^*^	0.090^a^	0.125^a^	0.151^a^	Benzene derivatives	0.369	4.563
37	1308	1-methylnaphthalene	0.076^a^	0.106^a^	0.080^a^	Benzene derivatives		
38	1400	Tetradecane^*^	0.457^a^	0.650^b^	0.656^b^	Hydrocarbons	0.316	2.364
39	1456	(E)-6.10.-dimethyl-5.9-undecadien-2-one	1.005^a^	0.934^a^	1.538^b^	Aldehydes and ketones		
40	1502	Pentadecane	0.547^a^	0.946^b^	0.536^a^	Hydrocarbons		
41	1520	Trichodiene^*^	0.010^a^	0.061^a^	0.031^a^	Terpenes	0.221	5.103
42	1529	Lilial^*^	0.007^a^	0.009^a^	0.033^a^	Terpenes	0.412	25.055
43	1597	Diethyl Phthalate	0.122^a^	0.249^b^	0.382^c^	Others		
44	1599	Hexadecane	0.524^a^	0.754^b^	0.660^b^	Hydrocarbons		
45	1999	Eicosane	0.283^a^	0.332^b^	0.530^c^	Hydrocarbons		
46	3007	Triacontane^*^	0.026^a^	0.057^a^	0.073^a^	Hydrocarbons	0.317	2.353
		Alcohols	30.17^a^	31.25^ab^	39.61^b^			
		Aldehydes and ketones	12.04^a^	8.12^a^	24.51^b^			
		Benzene derivatives	26.41^b^	33.15^c^	13.26^a^			
		Hydrocarbons	10.20^a^	16.85^b^	11.32^ab^			
		Terpenes	7.71^b^	13.45^c^	1.45^a^			
		Others	1.61^a^	9.51^c^	5.42^b^			

*Values in separate columns marked by the same letter (a, b, c) do not differ significantly at p = 0.05, according to HSD Tukey test*.

* *15 essential compounds based on stepwise discriminant analysis*.

*Wilks' lambda—values for compounds which were incorporated into the model on the basis of the performed stepwise linear discriminatory analysis for all the 46 identified volatile compounds. In “red” are compounds which have a highly significant effect on the differentiation of analyzed cereals*.

**Figure 3:**
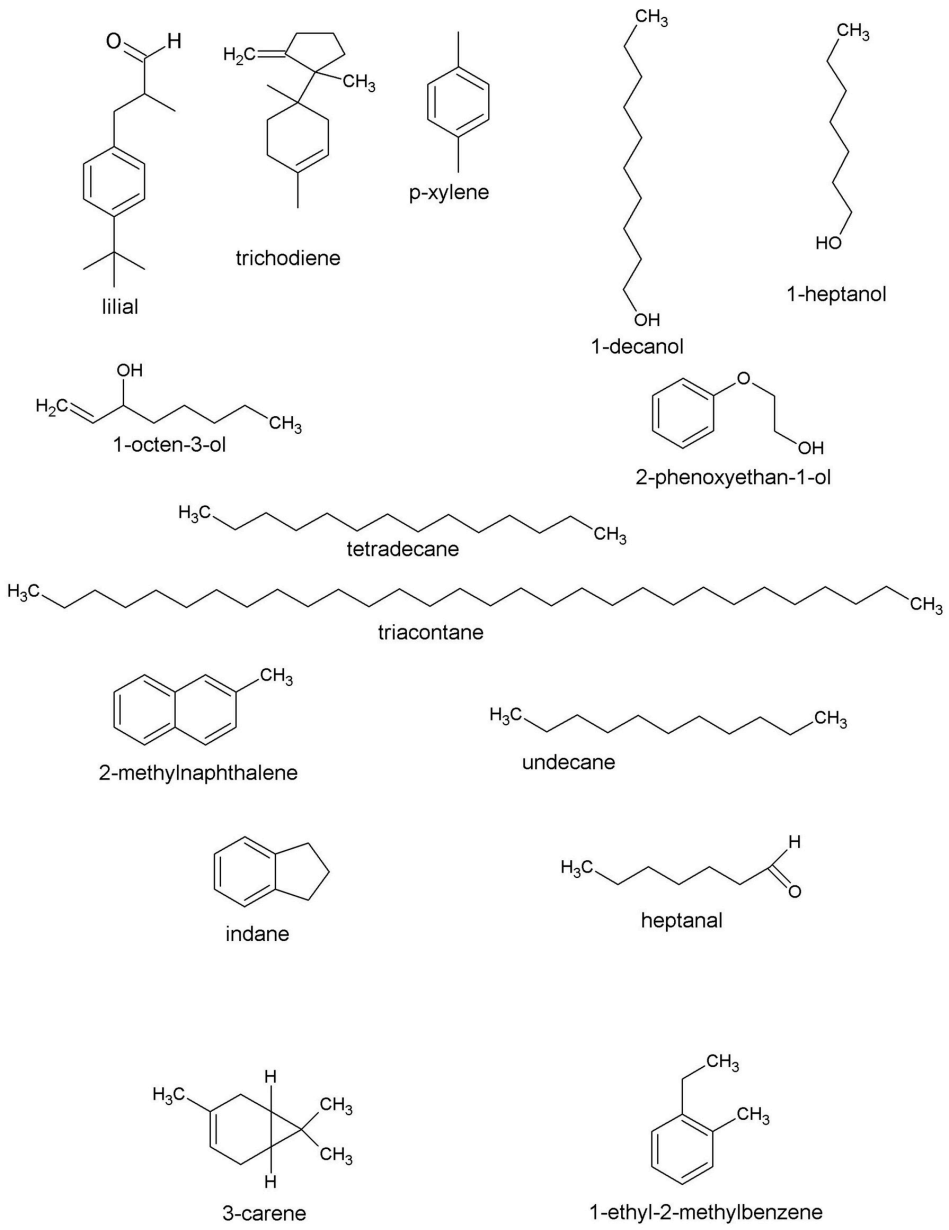
**Structures of volatile compounds included in model of SDA**.

**Figure 4:**
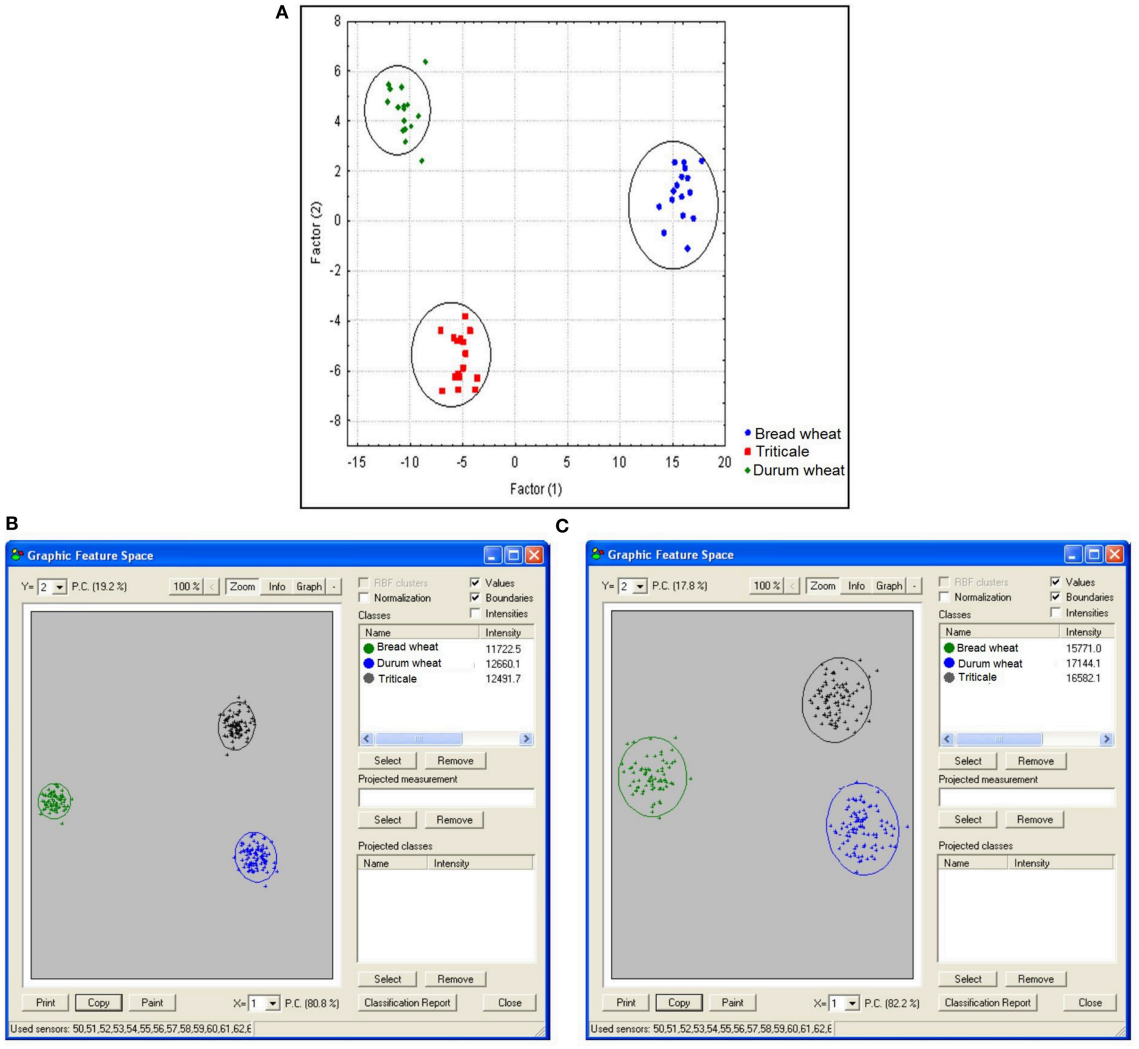
**Results of discriminatory analysis covering contents of volatile compounds identified in samples of bread wheat, durum wheat and triticale**. Wilks' lambda = 0.0003778. Function value test *F* = 0.8586911, calculated for this statistic at a probability level of *P* = 0.00001 **(A)**. A map for the range of masses of 50–250 **(B)**. A map for the range of masses of 50–150 **(C)**.

Tukey's test was carried out based on the results of the analysis of VOCs. From 201 compounds 15 VOCs (Figure 3) were significantly different in all grains. A subsequent stepwise discriminant analysis (SDA) was performed for only those 15 compounds (Figure 4A). Among them, the factors of greater power of discrimination were: lilial, trichodiene, and p-xylene. On the basis of the determined classification functions covering all the variables, test subjects were classified into groups concerned with correctness at different levels of ~90%.

**Figure 5:**
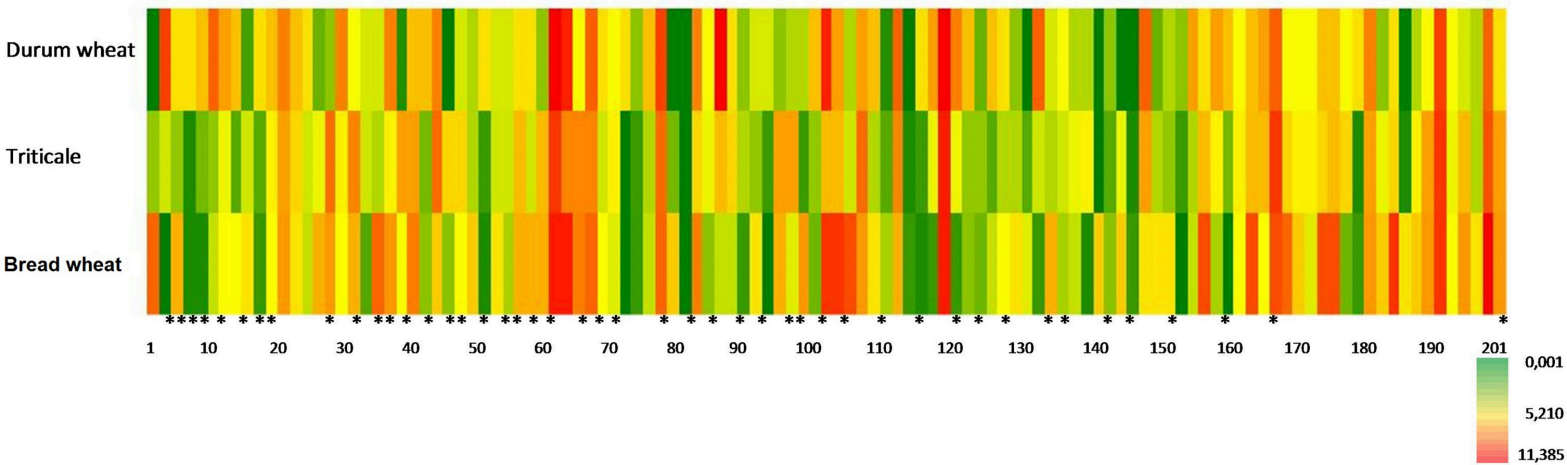
Heatmap of all volatile compounds determined from the concentrations given as relative units (RU) ^*^identified volatile compounds listed in Table 3.

Similarities and differences which characterized three cereal species suggested the advisability of conducting an analysis to separate these cereals depending on the percentages of combinations of retention indexes and ions. In order to visualize the relationships and differences in VOC levels for each sample, heatmaps (Figure 5) for metabolite profiles in new leaves were used.

To identify differences between the trial populations based on the concentration of volatile compounds, a comparative analysis of multi-dimensional objects and attributes was carried out. Detailed presentation of the distribution of important volatiles in the three analyzed cereals was accomplished with a heatmap. This helped to assess the extent to which the sample was characterized by the concentration of determined compounds. A similar color tone to the heatmap indicates the area—a group of samples—which based on the concentration of the analyzed compounds is homogeneous. This analysis clearly shows that the identified volatile compounds are discriminants under which objects can be assigned to the appropriate groups depending on the level of concentration of these compounds (fields marked ^*^ in the heatmap).

**Figure 6:**
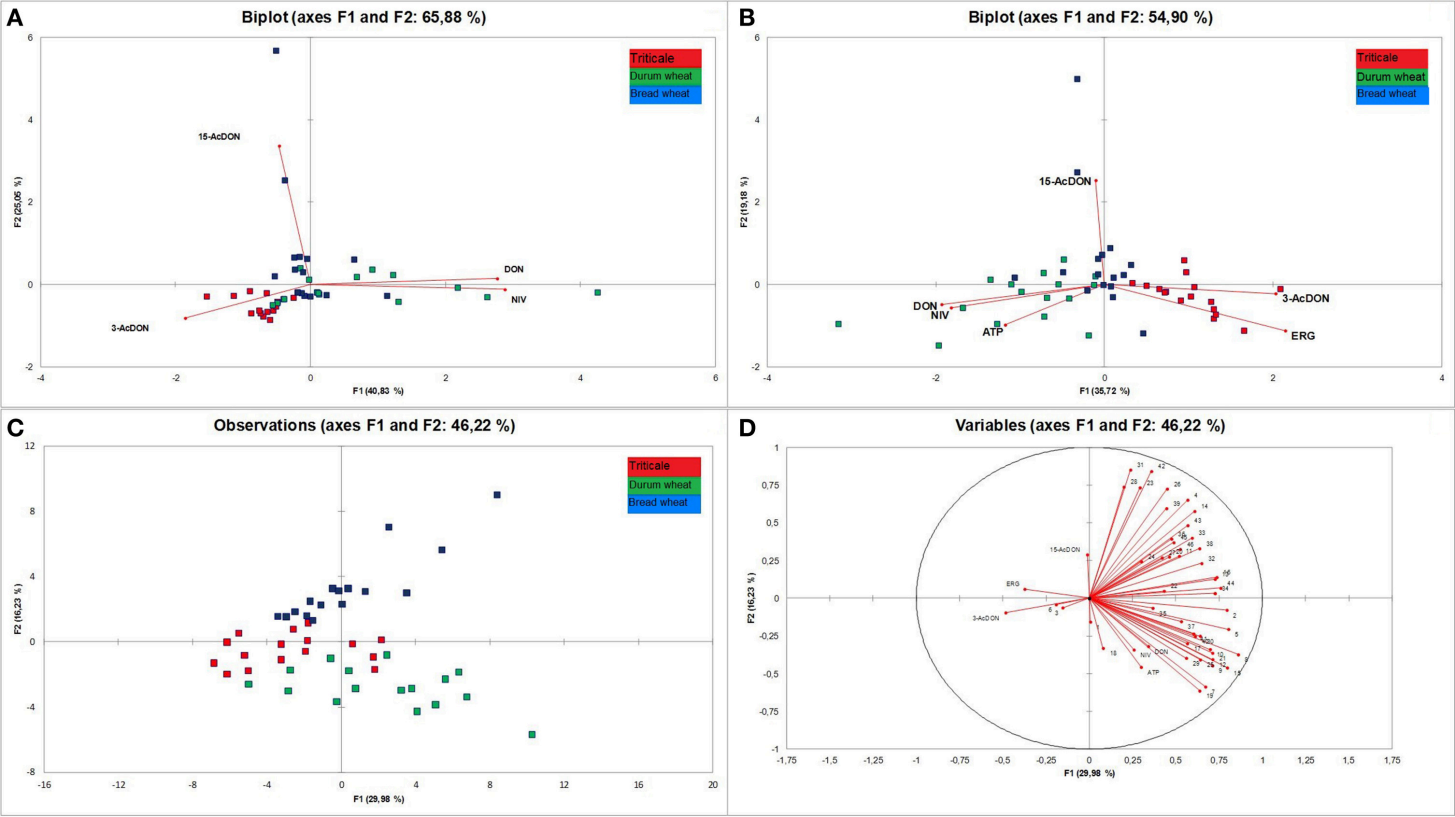
**Principal component analysis (PCA) biplots for trichothecenes of group B (A), trichothecenes, and fungal biomass indicators (ERG and ATP) (B) analyzed in 48 samples of cereal grain (triticale—red, durum wheat—green, and bread wheat—blue) and plot representing grain samples distribution (C) and correlation circle (D) of trichothecenes**. ERG, ATP, and VOCs (numbers correspond to compound names as in Table 3).

Based on these premises, the analysis of the three cereals was conducted. The aim of the modeled experiment was to comprehensively distinguish analyzed cereals by means of statistical analyses of the content of trichothecenes group B, ERG, ATP, and VOCs. In order to investigate the relationship between trichothecenes and the examined cereals we conducted PCA. The results are presented in Figure 6A. The first two principal components explained 65.88% of variability of the data. Based on the correlation circle, the first component (F1) was highly positively correlated with DON and NIV, and highly negatively correlated with 3-AcDON. The second component (F2) was highly positively correlated with 15-AcDON. Analysing observations in the same figure, where only the first two components are considered, some discrimination of cereals can be stated. Durum wheat samples tend to be located on the right part of the F1 axis, while triticale tends to be located on the left side of the F1 axis and the lower part of the F2 axis. Close to them but closer to the middle point the bread wheat samples are located. However, since the percentage of variability represented by the two factors is not very high (65.88%), to avoid a misinterpretation of the results, we decided to complement them. Analysing squared cosines of the observations, pointed to an important role of the F3 factor. Moreover, triticale samples, like the variable 3-AcDON, are located in the upper part of the F3 axis, above the F1/F2 plane, while bread wheat tends to be located in the lower part of the F3 axis, below the F1/F2 plane. Thus, considering the three factors (F1, F2, F3), better discrimination of cereals seems possible.

The analysis was then extended with an analysis of ERG and ATP (Figure 6B), which are fungal biomass indicators, and subsequently compared with the level of VOCs in grain. The distribution of variables was similar to that of mycotoxins only, but variables DON and NIV were supplemented with the variable ATP, and the variable 3-AcDON was accompanied by ERG. Such a set of variables resulted in a distribution of observations mainly along the F1 axis. Also, as in the case of previous PCA analysis, the location of triticale corresponded to 3-AcDON and ERG, and durum wheat to DON, NIV, and ATP. However, some durum samples, like most of the bread wheat samples, were close to the center. The last PCA was performed for all analyzed variables, i.e., trichothecenes, ERG, ATP, and VOCs (Figures 6C,D). Distribution of variables indicates that most of the VOCs have a stronger impact on the distribution of observations than DON, NIV, and ATP. In this case, durum wheat observations were located below the F2 axis and tended to be located on the right side as DON, NIV, ATP, and almost half of the VOC variables. Triticale observations were located mainly on the left side of the F1 axis but close to the F2 axis. Similarly located were 3-AcDON, ERG, dimethyl sulfone, and styrene, but VOCs were very close to the center. Bread wheat observations were situated above the F2 axis, and only some of them were on the right side of the F1 axis, where almost half of the VOCs were located. Generally, the distribution of observations suggests high diversity among each analyzed cereal group. Since the impact of VOCs on the distribution of observations was higher than that of trichothecenes, ERG and ATP, the pattern of distribution of observations for PCA for all variables is similar to PCA for VOCs only (figure not presented).

**Figure 7:**
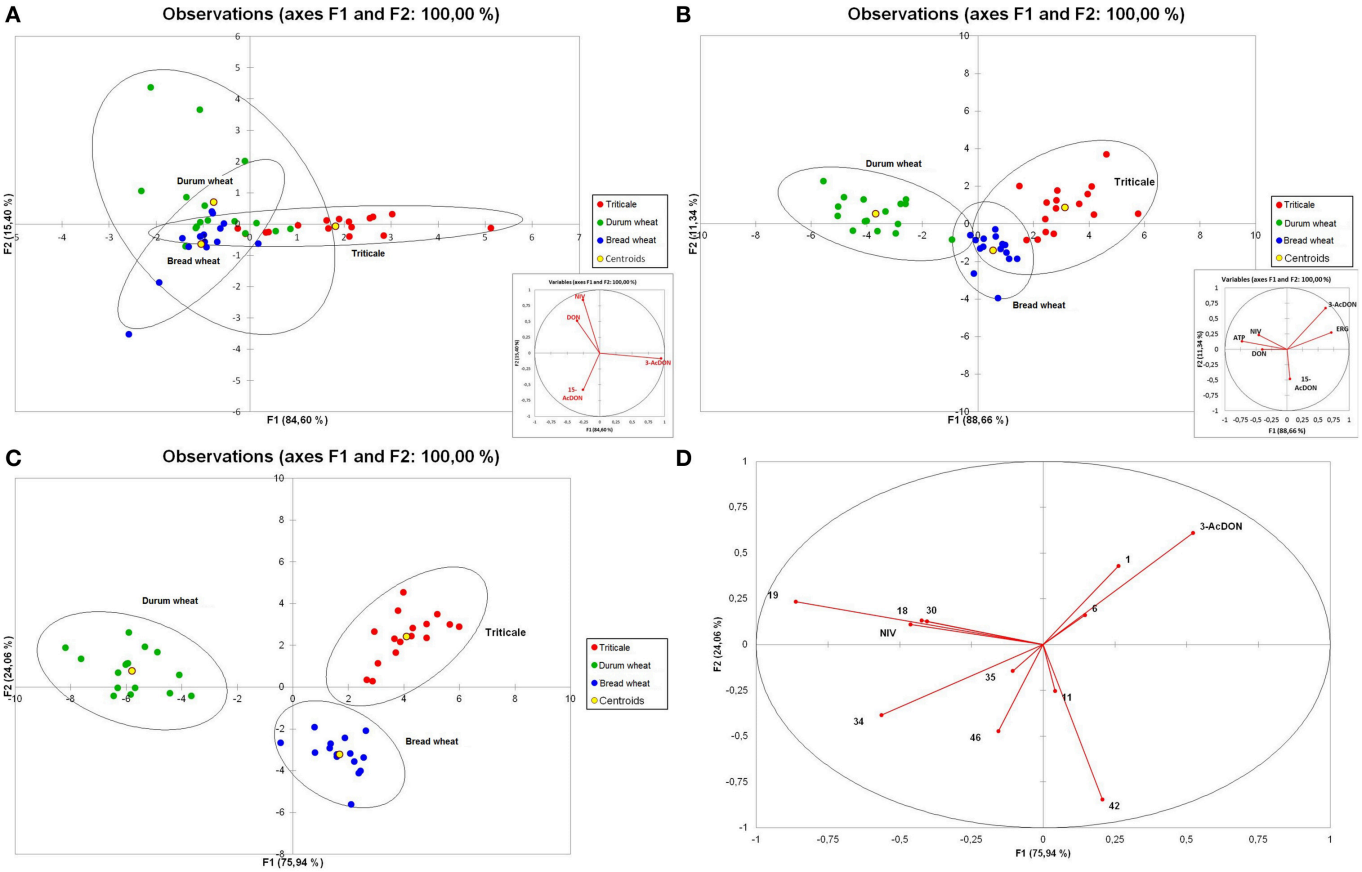
**Discriminant analysis (DA) for trichothecenes of group B—plot of observations and correlation circle for variables (A), trichothecenes and fungal biomass indicators (ERG and ATP)—plot of observations and correlation circle for variables (B) analyzed in 48 samples of cereal grain (triticale—red, durum wheat—green, and bread wheat—blue) and plot representing grain sample distribution (C) and correlation circle (D) of trichothecenes**. ERG, ATP, and VOCs (numbers correspond to compound names as in Table 2).

The subsequent analytical step after PCA was a discriminant analysis (DA) to determine the compounds that best separate the three cereal species. Initially, an analysis including trichothecenes was performed (Figure 7A). The inclusion of all four of the most important mycotoxins in the model allowed for 66.67% discrimination of cereals into groups. Extension of variables to incorporate fungal biomass markers, i.e., ERG and ATP, resulted in an increase of total discrimination up to 93.75% (Figure 7B). However, in this analysis the highest discriminant power was shown by 3-AcDON, ERG, and ATP. After completing the variables with VOCs, the obtained model allowed for the discrimination of analyzed cereals into groups at 97.92% (Figure 7C) since only one sample of triticale was posterior classified as bread wheat. As the forward selection method was applied, the model included the following compounds: limonene, lilial, 3-AcDON, 1-decanol, p-xylene, triacontane, NIV, cymene, dodecane, 1-heptanol, dimethyl sulfone, and nonanoic acid (Figure 7D).

**Figure 8:**
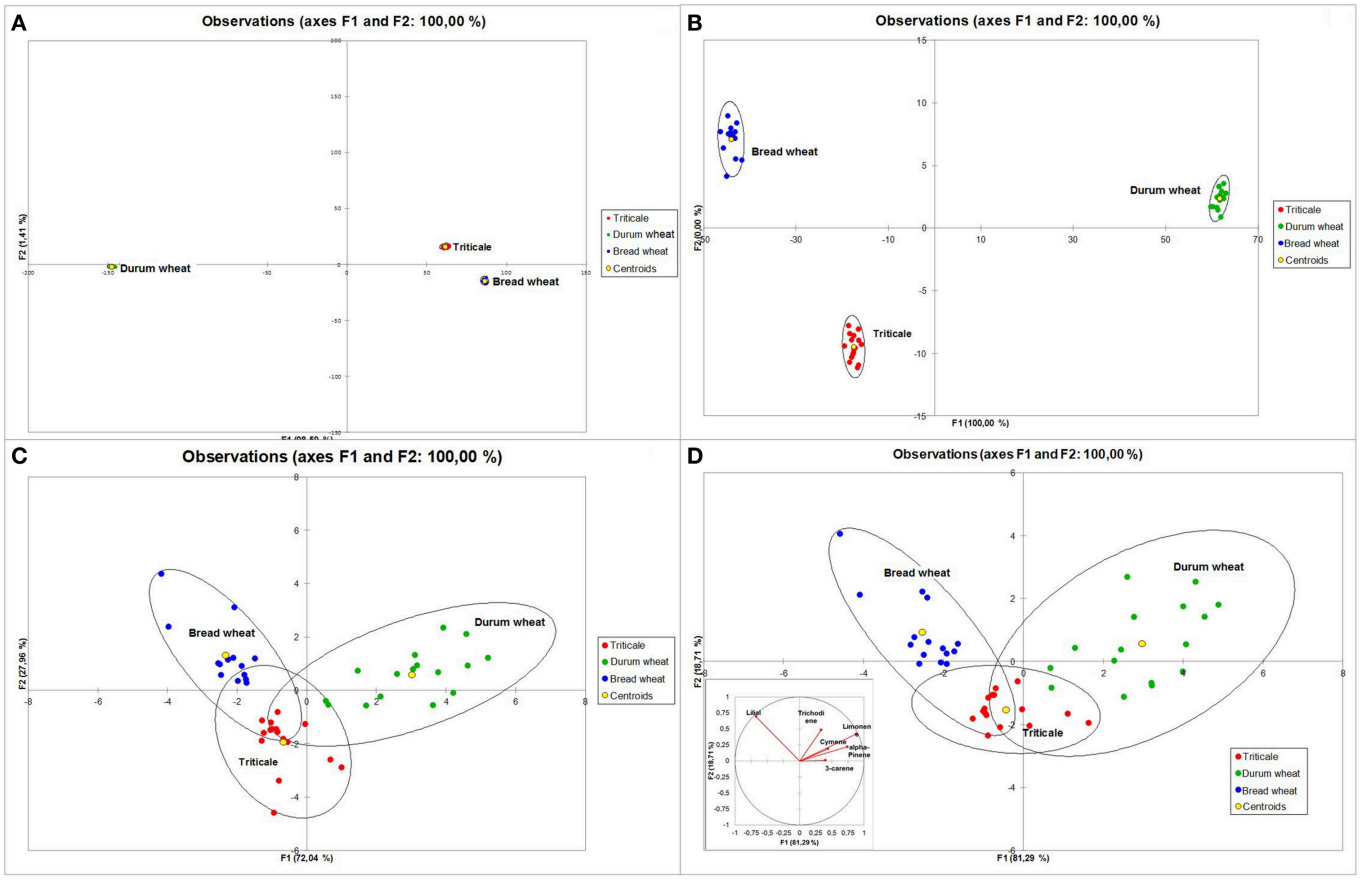
**Discriminant analysis (DA) for 46 identified VOCs—plot of observations (A), backward stepwise analysis—plot of observations (B), forward stepwise analysis—plot of observations (C) analyzed in 48 samples of cereal grain (triticale—red, durum wheat—green, and bread wheat—blue) and DA for terpenes—plot representing grain sample distribution and correlation circle (D)**.

The performed DA which took into account all 46 of the 201 generally identified VOCs, resulted in the complete discrimination of the analyzed cereals into three groups based on the first two factors F1 and F2 (Figure 8A). Triticale and bread wheat were discriminated by the F2 function, while the F1 function seems to discriminate mostly between durum wheat and triticale and bread wheat combined. Higher discriminant power was shown by: limonene, lilial, decanal, alpha-pinene, nonan-2-one, 1-decanol, benzene, nonanal, diethyl phthalate, and 3,5,5-trimethylcyclohexen-2-one. In order to find the most important variables in the model, a stepwise DA was performed. During backward stepwise analysis (Figure 8B) six compounds that contributed least to the prediction of group membership were eliminated (trichodiene, 5-butyldihydro-2(3H)-furanone, eicosane, 2-methylnaphthalene, dodecane, octanoic acid). In this case also 100% discrimination was obtained. The results of forward stepwise analysis (Figure 8C) on the other hand, allowed for a 95.83% total separation of the analyzed cereals. However, this was achieved with the inclusion of only the four following compounds: limonene, lilial, p-xylene, and indane. Since the group of compounds which contributed the most to separation was terpenes, an additional DA was carried out for this group (Figure 8D). In this case 95.83% discrimination was also obtained, as only two durum wheat samples were classified as triticale.

In the final step it was decided to verify the results recorded by the application of the electronic nose. This made it possible to confirm their previously performed differentiation in terms of volatile compound profiles. For each sample the spectrum was recorded within a range of 50–250 m/z. The obtained mass spectrum was statistically analyzed using PCA. In this analysis each of the obtained m/z values was a sensor. These sensors provided different contributions in separation of the data. Sensors which had the lowest contribution or no contribution could be eliminated from the analysis. Taking into consideration all the 200 sensors, triticale, durum wheat, and bread wheat were completely separated. The two principal components obtained in the course of this analysis explained 80.8% of differentiation (Figures 4B,C).

Thus, an attempt was made to further reduce the number of sensors in the above-mentioned range. In this case the level of contribution of individual ions in the separation of cereal into separate groups was taken into consideration. A narrowing of the PCA to only 100 ions from the 50–150 m/z range also made it possible to completely separate all the analyzed cereals.

## Discussion

In the framework of this study we analyzed three genetically similar grains: bread wheat, durum wheat, and triticale. Cereals are grown in the same ecological niche, under the same agronomic and climatic conditions. Accordingly, the environmental factors affecting the profile of fungal pathogens and the level of contamination from mycobiota as well as concentration of mycotoxins has been virtually eliminated. To date, field tests undertaken by other authors have often been subject to error as a result of the investigated crops being in different locations and under different conditions. It has been proved that weather conditions, especially rainfall during cereal flowering, have the most significant impact on the degree of pathogen infection and mycotoxin contamination level (Champeil et al., 2004). The research hypothesis of the present investigation assumed a discrimination of examinated cereals based on the profile of volatile compounds, the concentration of trichothecenes group B and contamination levels measured in the mycobiota using two chemical markers for three similar cereals. Chemometric methods were applied in order to determine the relationship between the individual test parameters determining the microbiological safety of cereal grains. The analysis of grain began to determine the mycobiota contamination level of whole grains (analysis of ERG) and the surface layer (analysis of ATP). The results indicate a low level of grain contamination. Previous studies by the authors (Perkowski et al., 2007) as well as those of other investigators (Maupetit et al., 1993) have shown that the level of ERG, together with the degree of mycobiota contaminated is characteristic of a particular cereal species and the average concentration was for grain of wheat 2–4 mg/kg; triticale 4–6 mg/kg; barley 7–8 mg/kg, rye 9–11 mg/kg oat 12–15 mg/kg (Perkowski et al., 2008b). In addition, an analysis of surface contamination was used by mean of ATP. This is a method commonly applied in the food industry, although it has not yet been widely adopted to analyse the surface contamination of cereals. Nevertheless, it was chosen due to the fact that the volatile compounds can be extracted from the surface layer of the kernel and therefore it was decided to check whether it might give results that better correspond with those for obtained volatile compounds.

Based on results determining the concentration of ATP and ERG it was found that a highly significant correlation coefficient for ERG and ATP was obtained only for a single species of grain. For all species of tested cereals no significant correlations were obtained, hence, it can be demonstrated that surface contamination of grain is related to the total infestation of the kernel, but only within individual cereal species. Pathogens are present among the native microflora of cereal grain in the field. We often see an infection of grain, and the presence of toxigenic strains of fungi of the genus *Fusarium* is associated with a mycotoxin produced of the group B trichothecenes at an early stage of pathogenesis. Therefore, it was decided to test a group B trichothecenes concentration in sample of cereal grains. In the climate of the Central Europe *Fusarium* fungi commonly dominate the ecological niche of pathogens of cereals (Logrieco and Visconti, 2004). Hence, it is necessary to analyse them for contamination by toxic metabolites which are trichothecenes. Among them, deoxynivalenol (DON) is the most common mycotoxin and is found in the highest concentrations. The above results would seem to indicate that low levels of contamination by trichothecenes were present in the analyzed samples of the cereal grains of the three species. In none of the tested samples were concentrations higher than the limit value indicated by the EU for the concentration of DON in cereals [Commission Regulation (EC) NO 1126/2007 of 28, September 2008] determined. Based on the results including levels of four mycotoxins DON, 3-AcDON, 15-AcDON, and NIV, it has been observed that in samples of triticale the results co-occurrence of DON concentrations and its acetyl derivatives differ to those of the other two wheats. Additionally, durum wheat was characterized by significantly higher concentrations of NIV. The results indicate a relationship of a quantitative profile of mycotoxins produced from cereal species. The studies of Montilla-Bascón et al. (2013) have revealed a similar relationship indicating that the same strains of toxinogenic pathogens growing on a variety of cereals may produce different amounts of mycotoxins, and even other mycotoxins. An important role is also played here by different chemotypes of particular species of microscopic fungi in the environment, as e.g.: in the case of *Fusarium graminearum* (Kulik, 2011). This is also related to the resistance of individual cereal species, and even to their varieties (Mesterhazy et al., 2005). During mycobiota infection of kernels compounds included in the cereal grain are broken down by microorganisms. A number of them are compounds belonging to the group of VOC's. Some of them are used in pathogenesis while others are metabolized by mycobiota, and some are metabolites of the pathogen or a native microflora. The VOC profile is therefore a mixture of compounds of different origin. One purpose of this work was to verify whether a diversity of VOCs exists even when cereals are genetically similar. The results were obtained using the two analytical methods; conventional gas chromatography coupled with a mass detector (TOF MS), and the application of an electronic nose. This combined approach has allowed the separation of the tested cereal species. It has been shown that they are characterized by a different profile of volatile compounds. It should be emphasized that as a result of the most commonly used statistical methods for this type of work (PCA and DA) metabolites were indicated to be the most differentiating tested cereal species. An important role was also played by different groups of VOCss, including terpenes. As part of our research we identified a number of compounds previously presented in the literature on the presence of VOCs in cereals. Among compounds which have discriminant power, special attention should be paid to trichodiene, observed in higher amounts in grain samples of inoculated small grain cereals (Jeleń and Wąsowicz, 1998; Demyttenaere et al., 2004) In the case of trichodiene, previous reports show that this compound was present in *Fusarium*-infected grain, since it is a volatile precursor in the trichothecene biosynthesis pathway (Jeleń et al., 1997b; Desjardins, 2006). At this point it was decided to verify the role of trichodiene, whether it may de facto differentiate individual species of cereals. To date it has been found that it differentiates cereals significantly but only those that are characterized by a higher degree of infestation with fungi from the genus *Fusarium*. In the analyses conducted by the authors of this study, when cereals were not significantly infested it was identified in all samples (Buśko et al., 2008).

An important observation is the lack of other sesquiterpenes in the volatile profile of the analyzed samples even though the spectrum of sesquiterpenes produced by fungi is very wide (Jeleń et al., 1995; Girotti et al., 2012; Kramer and Abraham, 2012).

Apart from trichodiene, higher amounts of other volatiles were observed in the course of fungal growth. The presence of 1-hexanol, limonene, pinene, nonanal, benzene, xylenes, and styrene was frequently reported (Korpi et al., 2009).

When investigating how volatile compounds are formed, it was found that microscopic fungi produce volatile compounds on the one hand as byproducts of metabolism under conditions adverse to their development. On the other hand, it was found that they may play the role of inhibitors of metabolic changes aimed at the adaptation of the microscopic fungi to the needs of the environment they are colonizing (Wilkins et al., 2003). The spectrum of volatile compounds identified to date includes alcohols, carbonyls and hydrocarbons. The primary volatile compounds identified in grain are 3-methyl-1-butanol, 1-octen-3-ol and other eight-carbon ketones and alcohols (Olsson et al., 2002; Demyttenaere et al., 2004).

Among these volatile compounds identified in samples, 1-hexanol is a characteristic compound produced by *Aspergillus clavatus* (Jeleń and Wąsowicz, 1998) and *Stachybotrys* (Wilkins et al., 2003), and high concentrations were determined in semolina and oat (Sides et al., 2001; Beleggia et al., 2009). Another compound found in tested samples was 2-ethyl-1-hexanol, which was identified by Wilkins et al. (2003) as a metabolite of fungi from the genus *Stachybotrys*. Fungi from the genus *Fusarium* produce 1,2,4-trimethylbenzene (Jeleń and Wąsowicz, 1998; Olsson et al., 2000), while α-pinene, limonene and propylbenzene are fungal metabolites (Jeleń and Wąsowicz, 1998; Beleggia et al., 2009) produced by *Penicillium, F. graminearum* (Jeleń et al., 1997a), and *Fusarium sporotrichioides* (Demyttenaere et al., 2003; Korpi et al., 2009).

Proposals for volatile compounds distinguishing individual grains have been supported by in-depth statistical analysis. However, further verification by mean of more and more often with positive results used in the laboratories of electronic nose, was performed (Buśko et al., 2014). Using 200 sensors achieved a complete separation into three groups cereals. It was assumed that reducing the number of sensors to 100 would be less effective. There have, however, been highly satisfactory results. The two methods of chemical analysis for determining the profile of VOCs supported by chemometric analysis clearly indicate that the three analyzed grains differ in their profile of volatile compounds. The use of methods including both volatile compounds, trichothecenes, ERG, and ATP allowed their exact role in the distribution of respondents cereals to be determined, together with their discriminatory strength. We hope that the results obtained will offer even a partial contribution to the study of pathogenicity in mycobiota, as well as a relevance for breeding work on obtaining varieties of these cereals resistant to environmental factors.

## Conclusion

Examination of 16 representative genetically similar genotypes of species of cereal (bread wheat, durum wheat, and triticale), grown under identical agro-meteorological conditions leveling of the environmental impact, led to a wider knowledge of group B trichothecenes and volatile compounds in the grain. Further, data on the associated with them mycobiota were obtained. It was found that the method used to study the ATP level was suitable only for individual groups of cereals, no such relationship was discovered for their total population. Toxin concentration was relatively low, the profile was similar but some variation of both concentration and co-occurrence was found among crops. The VOCs profile varied and allowed for their complete separation following the GC/MS analysis, which was fully confirmed by the electronic nose. Analysing using such chemometric methods as PCA and discriminant analysis, additional informations were obtained. The chemometric analysis allowed us to determine compounds (metabolites) (mainly volatile) characterized by the greatest discriminatory force. Among the discriminating compounds were those that have been described earlier in this type of analysis by other researchers, namely: lilial, trichodiene, and p-xylene.

The designed and applied research model, based on a comprehensive analysis of all the studied traits through their thorough statistical analysis, confirmed these observations and expanded the share of group B trichothecenes and mycobiota enriched inference. The results, we believe, will give rise to further work in this area and will probably be very helpful in cereal breeding with regard to their resistance to pathogen attack and environmental factors.

## Author contributions

MB, KS, HJ, JC, BT, JP designed the study, developed the methodology, collected the data, performed the analysis, and wrote the manuscript. TG designed the study, performed the field experiment, collected the data, and wrote the manuscript.

### Conflict of interest statement

The authors declare that the research was conducted in the absence of any commercial or financial relationships that could be construed as a potential conflict of interest.
